# Primary hyperparathyroidism characterized by diffuse homogeneous metastatic pulmonary calcification: A case report: Erratum

**DOI:** 10.1097/MD.0000000000013501

**Published:** 2018-11-21

In the article, “Primary hyperparathyroidism characterized by diffuse homogeneous metastatic pulmonary calcification: A case report”,^[1]^ which appears in Volume 97, Issue 44 of *Medicine*, the authors would like to note a change in author order. The author order should be Yuzhu Jia, Lihua Wang, Guangzhao Yang, Guoqun Mao, Yougen Cheng, Yulin Cao.
